# Beneficial Activities of *Alisma orientale* Extract in a Western Diet-Induced Murine Non-Alcoholic Steatohepatitis and Related Fibrosis Model via Regulation of the Hepatic Adiponectin and Farnesoid X Receptor Pathways

**DOI:** 10.3390/nu14030695

**Published:** 2022-02-07

**Authors:** Seung Ho Jeon, Eungyeong Jang, Geonha Park, Yeongae Lee, Young Pyo Jang, Kyung-Tae Lee, Kyung-Soo Inn, Jong Kil Lee, Jang-Hoon Lee

**Affiliations:** 1 Department of Fundamental Pharmaceutical Science, Graduate School, Kyung Hee University, 26, Kyungheedae-ro, Dongdaemun-gu, Seoul 02447, Korea; bawoojang@khu.ac.kr (S.H.J.); yalee88@khu.ac.kr (Y.L.); 2 Department of Internal Medicine, College of Korean Medicine, Kyung Hee University, 26, Kyungheedae-ro, Dongdaemun-gu, Seoul 02447, Korea; obliviona79@naver.com; 3 Department of Internal Medicine, Kyung Hee University Korean Medicine Hospital, 23, Kyungheedae-ro, Dongdaemun-gu, Seoul 02447, Korea; 4 Department of Life and Nanopharmaceutical Sciences, Graduate School, Kyung Hee University, 26, Kyungheedae-ro, Dongdaemun-gu, Seoul 02447, Korea; ginapark0326@khu.ac.kr (G.P.); ypjang@khu.ac.kr (Y.P.J.); ktlee@khu.ac.kr (K.-T.L.); 5 Department of Oriental Pharmaceutical Science, College of Pharmacy, Kyung Hee University, 26, Kyungheedae-ro, Dongdaemun-gu, Seoul 02447, Korea; 6 Department of Pharmaceutical Biochemistry, College of Pharmacy, Kyung Hee University, 26, Kyungheedae-ro, Dongdaemun-gu, Seoul 02447, Korea; 7 Department of Pharmaceutical Science, College of Pharmacy, Kyung Hee University, Seoul 02447, Korea; innks@khu.ac.kr

**Keywords:** *Alisma orientale*, fatty liver, steatohepatitis, adiponectin, farnesoid X receptor

## Abstract

The hepatic adiponectin and farnesoid X receptor (FXR) signaling pathways play multiple roles in modulating lipid and glucose metabolism, reducing hepatic inflammation and fibrosis, and altering various metabolic targets for the management of non-alcoholic fatty liver disease (NAFLD). *Alisma orientale* (AO, Ze xie in Chinese and Taeksa in Korean) is an herbal plant whose tubers are enriched with triterpenoids, which have been reported to exhibit various bioactive properties associated with NAFLD. Here, the present study provides a preclinical evaluation of the biological functions and related signaling pathways of AO extract for the treatment of NAFLD in a Western diet (WD)-induced mouse model. The findings showed that AO extract significantly reversed serum markers (liver function, lipid profile, and glucose) and improved histological features in the liver sections of mice fed WD for 52 weeks. In addition, it also reduced hepatic expression of fibrogenic markers in liver tissue and decreased the extent of collagen-positive areas, as well as inhibited F4/80 macrophage aggregation and inflammatory cytokine secretion. The activation of adiponectin and FXR expression in hepatic tissue may be a major mechanistic signaling cascade supporting the promising role of AO in NAFLD pharmacotherapy. Collectively, our results demonstrated that AO extract improves non-alcoholic steatohepatitis (NASH) resolution, particularly with respect to NASH-related fibrosis, along with the regulation of liver enzymes, postprandial hyperglycemia, hyperlipidemia, and weight loss, probably through the modulation of the hepatic adiponectin and FXR pathways.

## 1. Introduction

Non-alcoholic fatty liver disease (NAFLD) is an umbrella term frequently mentioned to cover a group of hepatic manifestations of non-alcoholic fatty liver (NAFL), non-alcoholic steatohepatitis (NASH), and NASH-related fibrosis [1]. Using the International Classification of Diseases, tenth revision (ICD-10) published by the World Health Organization (WHO) in 2019, three codes of K76.0 (Fatty (change of) liver, not elsewhere classified), K75.8 (other specified inflammatory liver diseases (NASH), and K74 (Fibrosis and cirrhosis of liver) may be commonly selected by clinicians to fill in the electronic medical records of NAFLD patients [2]. The shift from viral hepatitis to NAFLD in liver diseases is prevalent and is also accelerating. In addition, as the number of patients diagnosed with NAFLD increases, so does the risk of mortality. In particular, a cohort study in Sweden reported that reversing all stages of biopsy-proven NAFLD lowers the risk of overall mortality and extrahepatic cancer [3]. Hence, effective therapeutic strategies targeting various phenotypes of NAFLD, from simple steatosis to more serious forms of NASH and cirrhosis, are required to reduce the disease burden.

The increasing clinical need for the development of NAFLD drugs and attractive commercial opportunities has led to a large number of pipeline drug profiles [4]. Although a growing number of candidate drugs have been steadily investigated to halt or resolve NAFLD, to date, no US FDA-approved drugs have been found, mainly due to the complex pathogenesis of NAFLD, unsatisfactory effects not covering all aspects of the disease, and side effects [1]. Due to incompletely known mechanisms, clinicians frequently prescribe NAFLD patients with antioxidants such as vitamin E and omega-3 fatty acids, hepatoprotective agents including ursodeoxycholic acid, or metabolic agents such as insulin sensitizers or lipid-lowering drugs to regulate simple steatosis and NASH [5,6,7]. However, the use of these therapeutic options, though considered easily accessible by physicians, has some limitations, such as unsatisfactory effects at resolving NASH, unfavorable side effects, and inability to decrease the risk of comorbidities [1,4]. Hence, lifestyle modification via calorie restriction and exercise are the most routinely recommended and highlighted methods in NAFLD management. Nevertheless, there is still an urgent need to find effective and convenient medication for the condition, because of the difficulty of achieving long-term adherence to a healthy lifestyle.

While many kinds of herb-based therapeutic agents have been shown to have a relationship to NAFLD, *Alisma orientale* (Sam.) Juz. (AO) is a candidate with the potential to be developed as an anti-NAFLD drug. Currently, targeting nuclear receptors (FXR agonists, PPAR agonists, etc.) or modulating peptide hormones (growth hormone-releasing hormone, fibroblast growth factors, etc.) to ameliorate NASH has gained much attention, and drugs that do so are being developed [8]. To the best of our knowledge, there has been no study on the efficacy together with underlying mechanisms of hepatic FXR activation and adiponectin modulation of AO in improving NAFLD and metabolic features caused by exposing mice to a Western diet (WD) over a long period. Although preclinical studies investigating the efficacy against NAFLD and metabolic disorders in the presence of AO extract or Hugan Qingzhi tablets (AO 6 g, *Crataegus pinnatifida* 6 g, *Nelumbo nucifera* 4 g, *Typha orientalis* 3 g, and *Panax pseudoginseng* 1 g) including AO have been performed, there are some limitations regarding the use of murine models that have not fully progressed to advanced fibrosis after short-term feeding. In addition, some studies have insufficiently evaluated markers related to NAFLD [9].

We have previously reviewed the possibility of linking AO actions in NAFLD to hepatic FXR and adiponectin activation [10]. However, additional evidence is needed to confirm the underlying mechanisms of hepatic FXR and adiponectin, explaining the anti-NAFLD efficacy of AO. This is because of the limited data, such as only measuring serum adiponectin focused on obesity and gluconeogenesis without evaluation of hepatic fibrosis in mice fed a high-fat diet and AO extract for eight weeks [11], and evaluating the activation of FXR by AO extract in *α*-naphthylisothiocyanate-induced cholestasis in rat models and not NAFLD rodents [12].

Here, we investigated the pharmacological effects and underlying signaling pathways of AO extracts on a large spectrum of hepatic pathological lesions from simple steatosis to fibrosis, together with obesity, hyperglycemia, and hyperlipidemia, in mice exposed to a WD containing fat and sugar for 52 weeks. This is a relatively new model compensating for the shortcomings of giving only a high-fat diet to mice, as well as the methionine-and choline-deficient (MCD) dietary rodent models of NAFLD.

## 2. Materials and Methods

### 2.1. Materials

The dried rhizomes of AO were purchased from Kyung Hee Herb Pharm (Wonju, Gangwon Province, South Korea), a Good Manufacturing Product (GMP) compliant facility. Extra pure grade ethanol for extraction was acquired from Duksan Pure Chemicals (Seoul, South Korea). HPLC-grade acetonitrile and water were obtained from Fisher Scientific (Seoul, South Korea). Reference standards of alisol B, alisol B acetate, and alisol C acetate were purchased from Chemfaces (Wuhan, Hubei, China). Paraformaldehyde (PFA), DPX mounting medium, phosphate buffer (PB), tris-buffered saline (TBS), phosphate-buffered saline (PBS, pH 7.4), and Triton X-100 were obtained from Sigma-Aldrich (St. Louis, MO, USA). Protease/phosphatase inhibitor cocktail (78445) and RIPA buffer (89901) were acquired from Thermo Fisher Scientific (Waltham, MA, USA). Western diet (WD; D12079B) was obtained from Research Diets (New Brunswick, NJ, USA) Primary antibodies against FXR (72105S), adiponectin (2789S), phospho-AMPKα (2535 L), AMPKα (2532 L), phospho-FAK (3284S), FAK (3285S), phospho-Akt (4060S), and Akt (4685S) were purchased from Cell Signaling Technology (Danvers, MA, USA). Osteopontin (ab63856), CYP7A1 (ab65596), and F4/80 (ab111101) were obtained from Abcam (Cambridge, UK). Mouse anti-β-actin (sc-47778 HRP) and horseradish peroxidase (HRP)-conjugated secondary antibodies were acquired from Santa Cruz Biotechnology (Dallas, TX, USA). Goat anti-rabbit IgG (biotinylated), normal goat serum, and avidin-biotin complex (ABC) mixture were purchased from Vector Laboratories (Burlingame, CA, USA). Protein assay reagents, acrylamide, tetramethylenediamine (TEMED), and enhanced chemiluminescence reagents were obtained from Bio-Rad Laboratories (Hercules, CA, USA).

### 2.2. Preparation of AO Extract

The pulverized powder of the dried rhizome of AO was sieved with an 850 μm filter. The powder (300 g) was extracted with 3 L of 30% ethanol in an ultrasonic water bath (700 W, 40 kHz) at room temperature for 2 h. The extract was filtered through a filter paper (2.5 μm) under decompression conditions, concentrated on a rotatory evaporator under vacuum, and freeze-dried to produce AO extract powder (92.3 g, yield of 30.8%)

### 2.3. UHPLC-PDA-ESI-MS Analysis of AO Extract

For the liquid chromatographic mass analysis, the sample and the standard were prepared as follow: 10 mg of AO extract powder was dissolved in 1 mL of 30% ethanol for the sample solution; alisol B, alisol B acetate, and alisol C were each dissolved in 30% ethanol as the concentration of 1.0 mg/mL as the standard solutions. The sample and standard solutions were filtered through a 0.2 μm ployvinylidenefluoride (PVDF) syringe filter (Whatman, Maldstone, UK) just before being injected into the UHPLC system.

Liquid chromatographic analysis was conducted by a Waters Acquity H-class ultra-high performance liquid chromatography (UHPLC) system (Waters Corp., Milford, MA, USA) attached to a photodiode array (PDA) detector. An ACQUITY UPLC HSS T3 column (2.1 mm × 50 mm, 1.8 µm) united with an ACQUITY UPLC HSS T3 VanGuard Pre-column (2.1 mm × 5 mm, 1.8 µm) was utilized for chromatographic separation. The column oven temperature and sample storage temperature were maintained at 30 °C and 20 °C, respectively. The injection volume of the sample was 2.0 μL and the flow rate of the mobile phase was 0.35 mL/min. The gradient elution system was comprised of acetonitrile (solvent A) and water (solvent B) and controlled over time as follows: 1% A for 0–2 min, 1–10% A for 2–5 min, 10–20% A for 5–7 min, 20–70% A for 7–15 min, 70–90% for 15–20 min, 90–100% A for 20–25 min, 100–1% A for 25–26 min, and 1% A for 26–30 min. The selected detection wavelength was 210 nm. Mass spectrometric analysis was carried out on a JMS-T100TD (AccuTOF-TLC) spectrometer (JEOL Ltd., Tokyo, Japan) equipped with an electrospray ionization (ESI) source. The device settings of ESI-TOF-MS analysis in the positive ion mode were as follows: desolvating chamber temperature, 250 °C; orifice 1 temperature, 80 °C; scan range, *m*/*z* 50 to 1000; peak voltage, 1500 V; detector voltage, 2100 V; orifice 1 voltage, 40 and 80 V; orifice 2 voltage, 10 V; ring lens voltage, 5 V; and N_2_ gas flow rate, 1.0 L/min (nebulizing gas) and 3.0 L/min (desolvating gas). For the negative ion mode, the conditions of MS analysis were as follows: desolvating chamber temperature, 250 °C; orifice 1 temperature, 80 °C; scan range, *m*/*z* 50 to 1000; peak voltage, 1500 V; detector voltage, 2100 V; orifice 1 voltage, −80 V; orifice 2 voltage, −10 V; ring lens voltage, −5 V; and N_2_ gas flow rate, 1.0 L/min (nebulizing gas) and 3.0 L/min (desolvating gas). The noise spectrum was subtracted from the sample spectrum before the elemental composition was determined on selected peaks using the mass center main software (JEOL).

### 2.4. Animals and Treatment

Male C57BL/6 mice (seven weeks old) were purchased from Daehan Biolink Co., Ltd. (Eumseong, Chungcheiongbuk-do, Republic of Korea). Before the experiments, the mice were nurtured in ventilated plastic cages at a suitable temperature (23 ± 1 °C) and humidity (50 ± 10%) under a 12-h light/dark cycle. The mice were access to a normal diet (ND) and distilled water (DW) freely for one week to acclimatize to their environment. The mouse model of NASH was induced with WD and D-(−)-fructose (23.1 g/L)-D-(+)-glucose (18.9 g/L) dissolved DW treatment for 24 or 52 weeks. Stabilized animals were equally divided into three groups for 24-week experiment and four groups for 52-week according to body weight (24-week experiment: mice receiving ND, mice receiving WD, and mice treated WD with AO 250 mg/kg; 52-week: mice receiving ND, mice receiving WD, and mice treated WD with AO at 100 or 250 mg/kg; *n* = 12). After 12 or 26 weeks from the start of the experiment, AO extraction dissolved in DW and vehicle were orally administered three times per week for remain 12 or 26 weeks in each WD-fed group. All animal studies were conducted in accordance with the “Principles of laboratory animal care (National Institutes of Health publication number 80–23, revised 1996) and were approved by the Animal Care and Use Guidelines Committee of Kyung Hee University (approval number: KHSASP-20-591).

### 2.5. Glucose Tolerance Test

To examine alterations in glucose metabolism, an oral glucose tolerance test (OGTT) was executed. Before the test, the mice were starved overnight to create an empty stomach. The body weight of each mouse and fasting blood sugar were measured using a glucometer (Accu-Chek^®^ Active; Roche, Basel, Switzerland) before the test. The calculated glucose solution based on body weight (3 g/kg) was administered per os for each mouse. After 15 min, as time elapsed (until 120 min), blood glucose levels were determined with a glucometer in the same order of injection.

### 2.6. Liver Tissue Preparation

Mice were euthanized by administration of ketamine and xylazine mixture in saline (0.9% NaCl) as the anesthetic and after serum extraction, cardiac perfusion was performed immediately. For perfusion, 4% PFA in 0.1 M PB and PBS were used. After perfusion, the liver was enucleated, and its weight was measured. Then, the liver tissues were post-fixed with PFA at 4 °C. For the paraffin section, the liver tissues were paraffinised using a standard protocol. Using a microtome (Accu-Cut^®^ SRM™ 200; Sakura, Torrance, CA, USA), sequential 8-μm-thick tissue were sectioned and mounted on gelatin-coated glass slides.

### 2.7. Serum Analysis

Serum analyses were performed using SQLab (Yongin, Gyeonggi-do, Republic of Korea). Materials and kits used for the serum assay were purchased from Roche (Basel, Switzerland) and performed appropriately according to SQLab’s protocol.

### 2.8. Quantitative Real-Time Polymerase Chain Reaction

Quantitative real-time polymerase chain reaction (qRT-PCR) was prosecuted as described in our previous study [13]. Briefly, total RNA was sampled from the livers of mice and measured with spectrophotometer. RNA samples were converted to cDNA and diluted with nuclease free water to adjust the final concentration (250 ng). The cDNA was amplified and quantified by CFX Connect real-time PCR system (Bio-Rad Laboratories, Hercules, CA, USA).

### 2.9. Western Blot Analysis

Western blot analysis was performed as described previously [14]. Liver tissues were homogenized in RIPA buffer containing protease/phosphatase inhibitors (PPI). Protein samples (50 μg) were separated by SDS-PAGE with sodium dodecyl sulfate (SDS) sample buffer. Then, transferred to polyvinylidene difluoride (PVDF) membranes. Blocking solution (5% BSA in 0.1% Tween 20 contained TBS) was employed for 1 h at room temperature (RT). Then, blocking solution containing primary antibodies were incubated overnight at 4 °C. With washing buffer (0.1% Tween 20 in TBS), the membranes were washed three times for 5 min each. After then, horseradish peroxidase-conjugated secondary antibodies against mouse or rabbit IgG in blocking solution was incubated for 1 h at RT. The membranes washed with washing solution five times for 10 min was carried out using an ECL reagent (Bio-Rad Laboratories) for protein detection and visualized using ChemiDoc (Vilber, France). The quantification of intensity of the bands was measured by ImageJ software (National Institutes of Health, Bethesda, MD, USA).

### 2.10. Histopathologic Staining and NAFLD Activity Scores

Liver tissue sections were deparaffinized and subjected to conventional hematoxylin-eosin (H&E) and Picro Sirius Red (PSR) staining. Images of the sections were recorded using a BX51 immunofluorescence microscope (Olympus, Tokyo, Japan). To prove NASH severity, H&E-staining was performed with liver sections and assessed the NAFLD activity score (NAS), as done in previous studies [15]. The degree of steatosis (0–3), lobular inflammation (0–3), and ballooning degeneration of hepatocytes (0–2) were measured. The sum of the three categories, NAS, demonstrated the severity of NAFLD and/or NASH. The images were captured in three parts for each tissue, and the average score was used.

### 2.11. Immunohistochemistry

To remove endogenous peroxidase activity, deparaffinized sections were treated with 1% hydrogen peroxide for 15 min after PBS washing. The sections were blocked with PBS containing 0.3% Triton X-100 and normal goat serum for 30 min. After washing the tissues, rabbit anti-F4/80 (1:100) was incubated overnight at 4 °C and incubated with a biotinylated anti-rabbit IgG (1:200) for 1 h. The sections were gently incubated with ABC solution for another 1 h at RT. The color was developed using DAB for 2–5 min. The liver tissue embedded slide glasses were air-dried and cover-slipped using DPX histomount medium.

### 2.12. Enzyme-Linked Immunosorbent Assays

Enzyme-linked immunosorbent assays (ELISAs) of inflammatory cytokines, including TNF-α and IL-1β, were performed using a fluorescent-based ELISA kit (BD Biosciences, Franklin Lakes, NJ, USA) and appropriate standards according to the manufacturer’s protocol. The equal quantities of supernatants, which extracted with RIPA buffer containing PPI were analyzed. All inflammatory cytokine standards and experimental samples were run in duplicate, and used averaged results.

### 2.13. Statistical Analysis

All data were calculated using GraphPad Prism 8.0 software (Graph Pad Software Inc., San Diego, CA, USA) as the mean ± standard error of the mean (S.E.M.). Most results were analyzed using a one-way analysis of variance (ANOVA). Analysis requiring comparisons over time, including body weight and OGTT were using two-way ANOVA. Differences were considered statistically significant at *p* < 0.05.

## 3. Results

### 3.1. Identification of Phytochemicals in AO Extract

**Figure 1:**
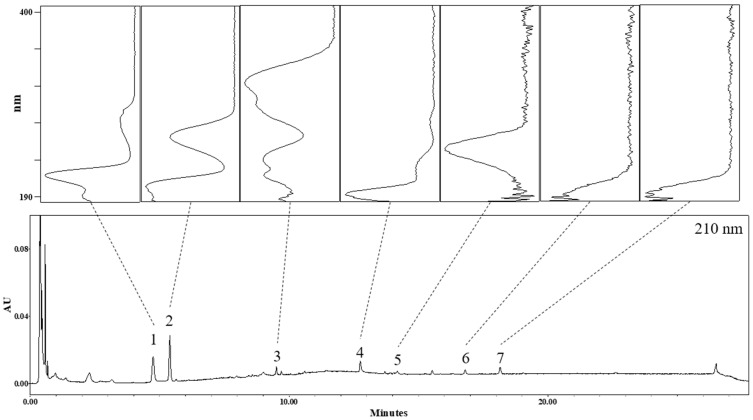
UHPLC chromatogram of the AO extract and UV spectrums of the seven peaks.

The UHPLC chromatogram of the AO extract was monitored at 210 nm, and the UV spectra of seven peaks are shown in Figure 1. Peaks 1 and 2 were identified as tryptophan and adenosine, respectively, by comparing their physicochemical data with those in the literature [16,17,18]. Peaks 5, 6, and 7 were confirmed as alisol C acetate, alisol B [19], and alisol B acetate, respectively, by direct comparison of the chromatographic profile with those of the corresponding reference standards. Peaks 3 and 4 were not confirmed as specified compounds; nonetheless, the molecular formula expected from the various adduct ions could be confirmed as C_26_H_30_O_8_ and C_30_H_46_O_5_, respectively. The detailed UHPLC-ESI-TOF-MS data for these seven peaks are listed in Table 1.

### 3.2. Administration of AO Extract Decreases WD-Induced Body and Liver Weight Gain and Prevented Hepatomegaly

**Figure 2:**
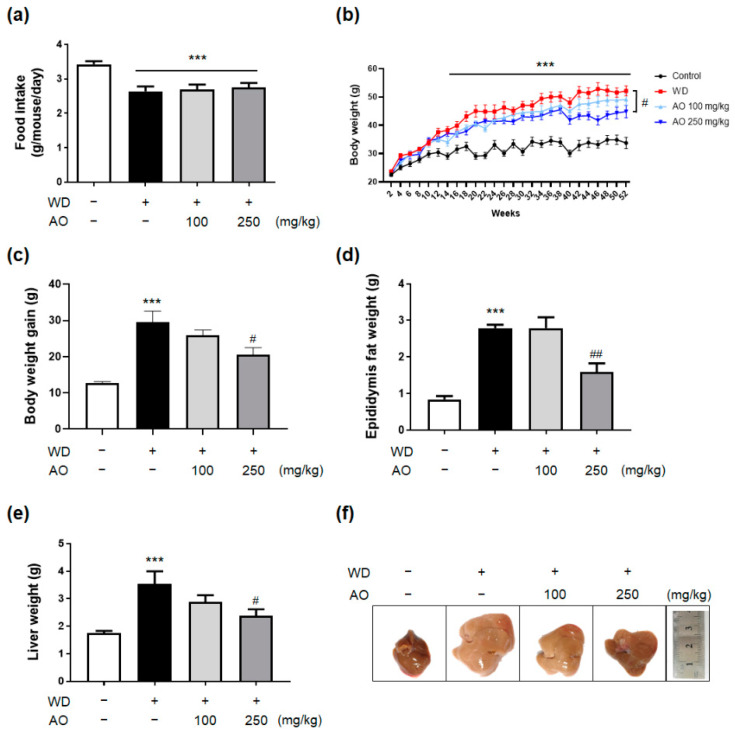
Administration of *Alisma orientale* (AO) extract decreases WD-induced body and liver weight gain and prevents hepatomegaly (**a**–**f**). (**a**) The food intake of mice was measured during the experiment period. (**b**) The body weight changes in mice following Western diet-induced nonalcoholic steatohepatitis (NASH). (**c**) The body weight gain was calculated. After the mice were sacrificed, (**d**) epididymis fat weights and (**e**) liver weights were measured. (**f**) Representative images of liver tissues. The data were analyzed by one-way analysis of variance (ANOVA) and (B) two-way ANOVA. *** *p* < 0.001 vs. control group; ^#^ *p* < 0.05, ^##^ *p* < 0.01 vs. WD-treated group (*n* = 12 per group). Plus sign (+) and minus sign (−) indicate the presence and absence of oral administration of WD or AO extract in male C57BL/6 mice, respectively.

As depicted in Figure 2a, no significant differences in the average amount of food that mice consumed for 52 weeks were observed among mice groups fed WD with or without administration of AO extract (100 and 250 mg/kg). Although the food intake of the control group was significantly higher than that of the others, mice fed a diet rich in fat and sugar and administered with vehicle or AO extract weighed significantly more than normal chow-fed controls from week 14 of WD feeding onwards (Figure 2b, *p* < 0.001 vs. the control group). Noticeably, a sustained weight reduction of 10% or more was observed in mice fed WD plus 250 mg/kg of AO extract from week 42 to week 52, and the intervention of AO extract (250 mg/kg) led to a significant decrease in the amount of body weight gain at week 52 compared to WD-induced mice (Figure 2c, *p* < 0.05, compared to the WD group). The phase of epididymal fat and liver weight loss achieved by the presence of 250 mg/kg of AO extract was similar to that of body weight loss (Figure 2d,e). In addition, mice exposed to WD diet for 52 weeks developed enlarged, heavy, and yellowish livers, representing higher hepatocyte lipid accumulation, but this distinguishable fatty infiltration and hepatomegaly were not found after AO extract treatment (Figure 2f). These results suggest that AO extract treatment reduces body weight gain and improves fatty and enlarged livers without a decrease in average food intake in the WD-induced mouse model.

### 3.3. Administration of AO Extract Improves Serum Liver Function, Lipid Profile and Glucose Level

**Figure 3:**
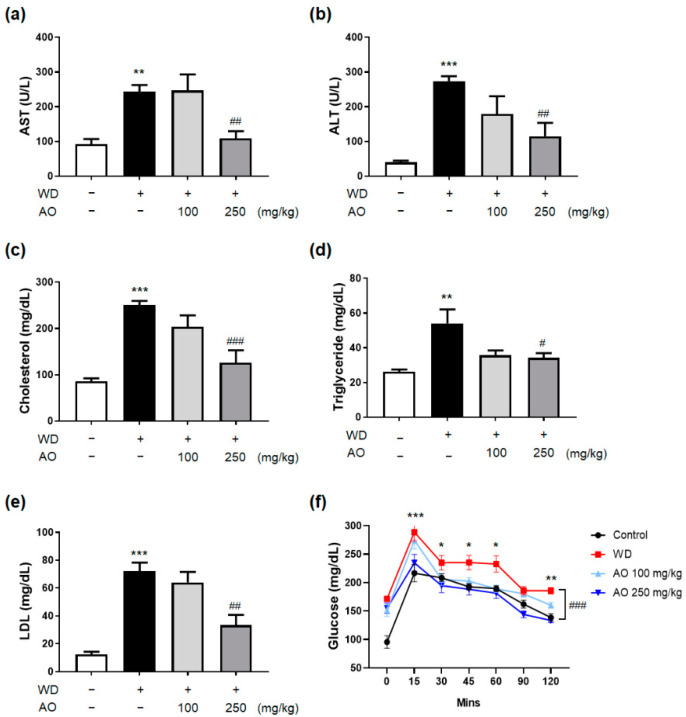
Administration of AO extract improves serum liver function, lipid profile, and glucose level (**a**–**f**). The levels of (**a**) alanine transaminase (ALT), (**b**) aspartate transaminase (AST), (**c**) cholesterol, (**d**) triglycerides (TG), and (**e**) low-density lipoprotein in serum. (**f**) Oral glucose tolerance test was assessed. * *p* < 0.05, ** *p* < 0.01, *** *p* < 0.001 vs. control group; ^#^ *p* < 0.05, ^##^ *p* < 0.01, ^###^ *p* < 0.001 vs. WD-treated group (*n* = 12 per group). Plus sign (+) and minus sign (−) indicate the presence and absence of oral administration of WD or AO extract in male C57BL/6 mice, respectively.

The increased body weight, liver weight, and hepatomegaly in WD-induced mice were accompanied by simultaneous elevation of basic diagnostic serum markers, indicating the presence of NAFLD and metabolic syndromes, such as serum AST, ALT, TC, TG, LDL, and glucose levels. The levels of serum AST and ALT, which are the most common biomarkers leaking into the circulation during hepatocyte necrosis in WD-induced mice receiving a 250 mg/kg dose of AO extract, were significantly decreased up to approximately 50% of those in the WD group (Figure 3a,b, *p* < 0.05, compared to the WD group). In addition, serum TC, TG, and LDL levels were markedly reduced by 250 mg/kg AO extract administration (Figure 3c–e). Interestingly, serum glucose levels dramatically decreased up to (15, 45, 120 min) or even lower (30, 60, 90 min) than those of the normal group when 250 mg/kg of AO extract was added (Figure 3f). This suggests that AO extract might be effective in responding sensitively to the abnormal elevation of blood sugar levels.

### 3.4. AO Extract Intervention Resolves Histological Injury of NASH with Fibrosis

NASH encompasses liver injury that represents a wide array of intrahepatic histological disruptions, as well as hepatomegaly, body weight gain, and abnormalities in serum markers. In particular, hepatic inflammation and fibrosis are important histological features of NASH that can trigger extensive liver damage, eventually resulting in accelerated NASH progression and the initiation of carcinogenesis [20]. Therefore, improvements in liver tissue in subjects with NAFLD implicate beneficial actions in the context of pharmacotherapy. Many clinical trials and studies on biomarker development have focused on improvement of the histological component of the disease [21].

**Figure 4:**
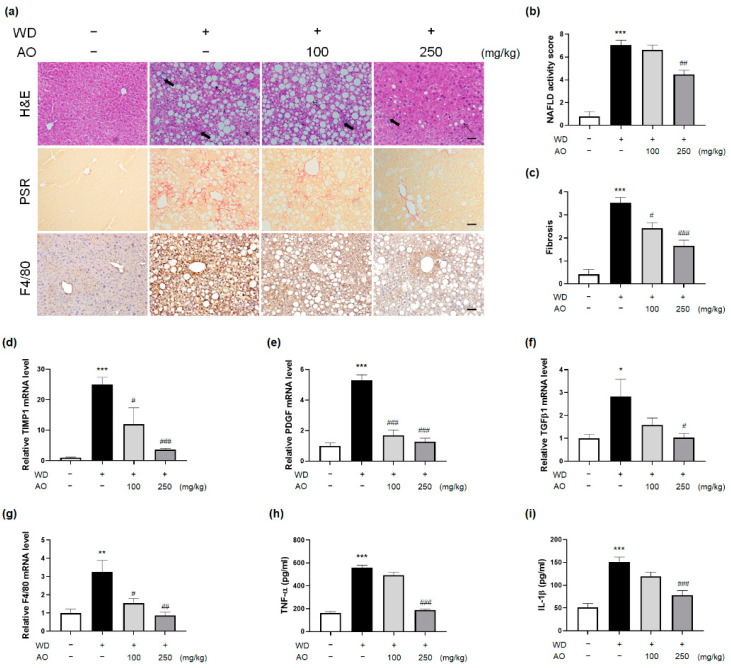
AO extract intervention resolves histological injury of NASH with fibrosis (**a**–**i**). (**a**) Representative images of H&E, picro sirius red (PSR) (*n* = 6 per groups), and F4/80 staining (*n* = 4 per groups) of mice liver tissues (scale bar = 50 μm; arrow: steatosis; bold arrow: lobular inflammation; dotted line arrow: ballooning). (**b**) NAFLD activity scores and (**c**) fibrosis scores were evaluated. The mRNA expression of (**d**) TIMP metallopeptidase inhibitor 1 (TIMP1), (**e**) platelet-derived growth factor (PDGF), (**f**) transforming growth factor beta 1 (TGFβ1), (**g**) F4/80, and the protein production of (**h**) tumor necrosis factor-α (TNF-α) as well as (**i**) interleukin-1β (IL-1β) in the liver (*n* = 6 per group) were evaluated. * *p* < 0.05, ** *p* < 0.01, *** *p* < 0.001 vs. control group; ^#^ *p* < 0.05, ^##^ *p* < 0.01, ^###^ *p* < 0.001 vs. WD-induced group. Plus sign (+) and minus sign (−) indicate the presence and absence of oral administration of WD or AO extract in male C57BL/6 mice, respectively.

To investigate the pharmacological effects of AO extract against NASH-related histological injury, we performed image analysis of liver tissue sections using H&E staining for quantitative analysis. In normal mice, no steatotic and inflammatory changes were observed in these staining analyses, while WD-induced mice displayed pronounced deposition of lipid droplets, lobular inflammation, and hepatocyte ballooning. In contrast to the WD-induced group, administration of 250 mg/kg dose of AO extract reversed liver histological changes by reducing the number of clear lipid vacuoles, inflammatory foci, and enlarged and rounded hepatocytes (Figure 4a). This intervention resulted in a significant decrease in the NAFLD activity score (NAS), which is the sum of scores for histological features (Figure 4b,c, Table 2, *p* < 0.05, vs. the WD group).

As shown in Figure 4a, slow but severe fibrogenesis was a notable histological change in mice induced with WD for 52 weeks compared to normal mice. Evidently, treatment with AO extract (250 mg/kg) ameliorated the increased PSR-positive tissue and fibrosis score in WD-induced mice with hepatic fibrosis (Figure 4a,c, Table 2, *p* < 0.05, vs. the WD group). In accordance with the results of immunohistochemistry, the hepatic mRNA expressions of fibrosis mediators, such as TIMP-1, PDGF, and TGF-β1, as assessed by qRT-PCR, were significantly suppressed by the intervention of 250 mg/kg dose of AO extract (Figure 4d–f). These improvements corresponded to the changes in NAS scores.

Kupffer cells, the main liver macrophages, play a crucial role in the onset and progression of NASH, with the depletion of these hepatic macrophages able to ameliorate NASH [22]. Compared to mice maintained on WD, mice administered with 250 mg/kg dose of AO extract showed remarkably decreased relative F4/80 expression. Notably, the infiltration of hepatic F4/80-positive cells was reduced to that of the control group after AO extract (250 mg/kg) treatment (Figure 4a,g). Moreover, hepatic TNF-α and IL-1β protein expression was also decreased by the presence of the AO extract (250 mg/kg) (Figure 4e,f). Collectively, the above findings suggested that AO extract administration significantly improved the pathophysiology related to NASH, which also implied that the promising efficacy of herbal plants may extend to treat more advanced stages of NAFLD.

### 3.5. Administration of AO Extract Affects Adiponectin and FXR Activation

**Figure 5:**
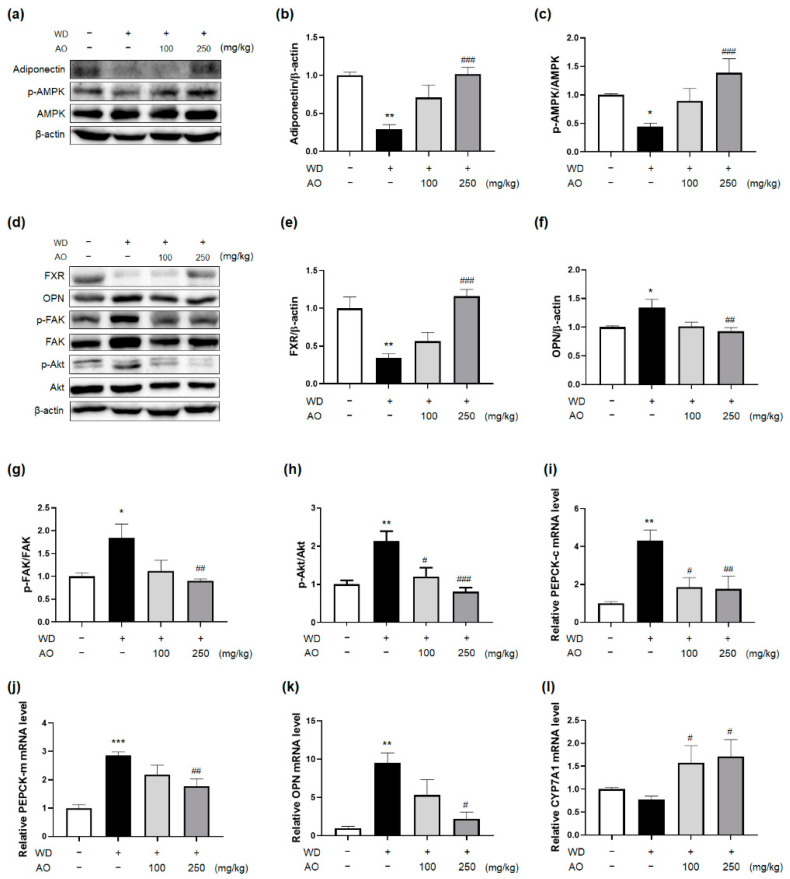
Administration of AO extract affects adiponectin and farnesoid X receptor (FXR) activation. (**a**) Representative immunoblotting images and quantitative protein expressions for (**b**) adiponectin and (**c**) AMP-activated protein kinase (AMPK) in mice liver. (**d**) Representative immunoblotting images and quantitative protein expressions for (**e**) FXR, (**f**) osteopontin (OPN), (**g**) focal adhesion kinase (FAK), and (**h**) protein kinase B (AKT). The mRNA expressions of (**i**) phosphoenolpyruvate carboxykinase-cytosolic (PEPCK-c), (**j**) PEPCK-mitochondrial (PEPCK-m), (**k**) OPN, and (**l**) CYP7A1 in the mice liver. * *p* < 0.05, ** *p* < 0.01, *** *p* < 0.001 vs. control group; ^#^ *p* < 0.05, ^##^ *p* < 0.01, ^###^ *p* < 0.001 vs. WD-treated group (*n* = 6 per group). Plus sign (+) and minus sign (−) indicate the presence and absence of oral administration of WD or AO extract in male C57BL/6 mice, respectively.

The protein levels of hepatic adiponectin and FXR, which are in charge of NASH-related inflammation, fibrosis, and glucose and lipid metabolism disorders, were significantly suppressed in WD-induced mice, while receiving AO extract led to a dose-dependent enhancement (Figure 5b,e). In particular, adiponectin plays a critical role in the regulation of hepatic glucose and lipid metabolism. Specifically, adiponectin-AMP-activated kinase (AMPK) activation accelerates lipid expenditure and prevents TG accumulation in hepatocytes [23]. In addition, adiponectin-phosphoenolpyruvate carboxy kinase (PEPCK) signaling regulates gluconeogenesis [24]. To investigate whether AO extract has an impact on the adiponectin pathways, the mRNA levels of hepatic PEPCK-c and PEPCK-m were analyzed using qRT-PCR. The administration of 250 mg/kg of AO extract reversed the increased mRNA expression of hepatic PEPCK-c and -m induced by WD maintenance (Figure 5i,j). Next, the effect of AO extract on FXR downstream was assessed by evaluating osteopontin (OPN), FAK/AKT, and CYP7A1 expression in the liver tissue of the present models. FXR activation often counteracts OPN expression, resulting in the suppression of FAK/AKT and induction of CYP7A1 liver expression [25], which eventually improves NASH-associated liver injury. As expected, there were statistically significant alterations in FXR-OPN-FAK/AKT protein levels in the hepatocytes of the AO extract (250 mg/kg)-treated mice (Figure 5d–g). The mRNA expression of OPN and CYP7A1 was also regulated, as expected (Figure 5k,l). Taken together, these results suggest that AO extract-induced proper regulation of both signaling pathways that are altered in NAFLD pathology may contribute to the beneficial actions of the plant.

## 4. Discussion

For the past several decades, a large number of studies have been performed to unveil the various prophylactic and therapeutic activities of herbal medicines that have been safely used for thousands of years against NAFLD pathogenesis [9]. AO, an herbal plant belonging to the family *Alismataceae,* has received a lot of attention in recent years. Here, we showed, for the first time, that 26 weeks of treatment with AO extract alleviated NAFLD-related conditions, along with obesity, hyperlipidemia and hyperglycemia through two key mechanisms of FXR and adiponectin activation in WD-induced mice adjusted to closely mimic the whole spectrum of human NAFLD pathophysiology as accurately as possible.

Previous clinical studies showing time of NAFLD progression have suggested that it takes about 3–7 years for simple steatosis to become NASH [26] and about 10–20 years for NASH to develop fibrosis [27]. Actually, we performed the 24-week preliminary experiment with similar protocol (three times administration per week for 12 weeks) before this study. In the preliminary study, we found positive effects in serum analysis such as LDL, cholesterol, AST, ALT and TG level, but the liver weight and body weight did not show any differences after AO treatment (Supplementary Figure S1). Therefore, such a long period of 52-week evaluation may be of particular need in our research because NASH and fibrotic changes in hepatic tissue occur in association with steady intake of WD. Instead, the actual administration period of AO extract was the latter 26 weeks in our study, while WD was administered for 52 weeks. Moreover, adding D-(-)-fructose (23.1 g/L)-D-(+)-glucose (18.9 g/L) to WD can induce significant hepatic fibrosis [28,29] with metabolic syndromes [30] as well as simple steatosis. Our mice model may also comprehensively mimic metabolic associated fatty liver disease (MAFLD) conditions that better reflect roles of underlying metabolic disorders driving NAFLD progression [31].

After 52 weeks of WD intake, C57BL/6 mice showed NASH-like features of severe steatohepatitis and fibrosis with high NAS, weight gain, hyperglycemia, and hyperlipidemia. Administering WD-induced mice with 250 mg/kg of AO extract from week 26 to week 52 reversed the biochemical and histological abnormalities associated with NAFLD, thus improving the components of NAS and fibrosis grading. Specifically, administration of AO extract (250 mg/kg) significantly inhibited the influx of F4/80 macrophages, reduced the secretion of inflammatory cytokines (TNF-α and IL-1β), and decreased the mRNA expression of pro-fibrogenic genes (TGFβ1, PDGF, and TIMP-1) in the hepatic tissue of mice fed WD. Although further data need to be obtained in isolate hepatocytes from the liver or in culture cells to determine specifically which cells in the liver AO extract affect, it is worth noting that treating AO extract resulted in a marked 70–80% decrease in fibrogenic gene expression upregulated by WD maintained for 52 weeks. This suggests that AO can be a promising option containing active pharmaceutical constituents capable of halting or reversing fibrosis, or achieving the resolution of NASH, which is the key therapeutic goal of NAFLD pharmacotherapy. As we previously reported, AO extract might exert anticancer effects against liver cancer, in addition to attenuating hepatic inflammation and fibrosis [32]. However, no animal-based evidence on inhibition of tumor growth by AO extract was found because our WD-induced mice did not develop NASH-associated hepatic carcinogenesis at 52 weeks after initiation of the diet.

Since metabolic syndromes, such as obesity, diabetes, and dyslipidemia, partly share the pathologic mechanisms of NAFLD and influence its progression to advanced liver diseases, we evaluated whether administration of AO extract effectively contributed to metabolic regulation [33]. After 26 week-treatment with AO extract (250 mg/kg), liver and body weight gain, as well as epididymal fat weight, showed a significant decrease in overweight mice fed WD for 52 weeks. Consistent with clinical data showing the relationship between weight loss and histological resolution in NASH patients, over 10% body weight loss and improved histological findings occurred in mice fed 250 mg/kg of AO extract added to WD. Weight reduction by AO extract was not accompanied by a decreased appetite, indicating that AO may be involved in energy expenditure or glucose and lipid metabolism. In particular, hyperglycemia and hyperlipidemia are closely associated with NAFLD development and progression [34,35]. When WD-induced mice are treated with AO extract (250 mg/kg), these metabolic disorders related to obesity and NAFLD improve, and levels of serum glucose, TC, TG, and LDL decreased significantly.

Since diabetes and dyslipidemia are common components of metabolic disorders frequently observed in NAFLD patients, and antidiabetic and lipid-lowering drugs, such as TZD, metformin, and statins, play an important role in managing hyperglycemia and hyperlipidemia and lowering the risk of cardiovascular disease in these patients. Although metformin and statins ameliorate insulin resistance and inhibit hydroxyl-methyl-glutaryl-coenzyme A reductase, respectively, they are no longer recommended as medications for treating NAFLD by the American Association for the Study of Liver Diseases (AASLD)/European Association for the Study of Liver (EASL) for the following reasons [36]: metformin, an AMPK activator, might induce hepatic cholestasis and idiosyncratic hepatotoxicity [37]. Statins, despite having more pros than cons, have been reported to elevate aminotransferase levels, leading to possible hepatotoxicity [38]. With regard to TZD (PPARγ agonists), their use for treating NAFLD has been reported to improve steatosis, inflammation, fibrosis, and insulin resistance, and the AASLD and EASL guidelines recommend their use in patients with NASH [36]. However, TZD still has adverse effects, including increased fluid retention, body weight, bone loss, and elevated risk of fracture and bladder cancer [36].

TZD-induced weight gain may be attributed to increased PEPCK enzyme levels in adipocytes, thus activating free fatty acid re-esterification and eventually resulting in weight gain [39]. In contrast, the AO extract (250 mg/kg) decreased the hepatic expression of PEPCK mRNA and inhibited hepatic gluconeogenesis. The effect of AO extract on hepatic glucose metabolism did not lead to weight gain, and showed beneficial effects on all NAFLD-related conditions as TZD did. As upstream targets of the PEPCK gene, hepatic adiponectin levels and AMPK activation were significantly increased by AO extract (250 mg/kg). Decreased levels of hepatic adiponectin expression may be closely associated with advanced stages of inflammation and fibrosis in obese NAFLD patients [40]. Exposure to AO extract resulted in a dose-dependent increase in adiponectin protein production in the liver tissue of mice fed WD. These findings are supported by the decreased expression of TNFα protein following AO extract administration in the liver, because hepatic adiponectin induction may reduce NASH-related necro-inflammation and fibrosis via TNF-α antagonism and suppress each other’s secretion [41]. AMPK is a critical signaling pathway involved in inhibiting hepatic de novo lipogenesis and increasing fatty acid oxidation and insulin sensitivity, but the controversial role of this signaling in AMPK-FXR crosstalk, which induces cholestasis and hepatotoxicity, has been reported [37,42]. Administering WD-induced mice with AO extract (250 mg/kg) also enhanced the phosphorylation of AMPK in the liver, further highlighting that its extract exerted beneficial effects on bile acid excretion from the liver via FXR downstream signaling. FXR acts as a multi-targeted regulator that interacts with hepatic inflammation, fibrosis, and metabolic disorders of glucose, lipids, and energy [43]. AO extract (250 mg/kg) significantly reversed the suppression of hepatic FXR protein production by WD for 52 weeks; indeed, its treatment improved the pathological manifestations of NAFLD and metabolic syndrome. Hepatic FXR activation by AO extract inhibited OPN in liver tissue, and then inactivated FAK and AKT, as demonstrated by ELISA analysis. Sequentially, hepatic CYP7A1 mRNA levels, a protein-coding gene involved in cholesterol efflux in the form of bile acids from hepatocytes, were significantly elevated in mice consuming AO extract (250 mg/kg). The signaling underlying the efficacy of AO extract is similar to that of obeticholic acid, a well-known FXR agonist, in that both activate hepatic FXR expression, but there is a slight difference between them in terms of the mechanisms regulating CYP7A1 activity. While activation of FXR by obeticholic acid represses the level of CYP7A1 metabolizing cholesterol to bile acids, leading to increased levels of serum LDL and itching, AO extract elevates hepatic expression of the enzyme and lowers serum LDL levels.

Although the specific and elaborate mechanisms involved in the anti-NAFLD effects of AO extract still remain to be studied in another NASH model or in rodent models fed high-fructose alone for enhanced evidence of efficacy, we suggest that adiponectin-AMPK, together with FXR-OPN-FAK/AKT regulation in hepatocytes, might be involved in the disease’s underlying signaling pathways. Further studies are needed to determine whether AO extract intervenes in the enterohepatic circulation of bile acids, since FXR is highly expressed in the intestine and hepatic tissues. In addition, confirming the effects of AO extract on gut microbiota, bile acid homeostasis, oxidative stress, carcinogenesis, and FXR-related metabolic proteins in NAFLD might be helpful in determining the utility of AO extract as an anti-NAFLD drug. Furthermore, in order to accumulate encouraging results based on more thoroughly planned animal studies, and consequently show promising outcomes in clinical trials, the interest and continuous investigations on AO extract that work against NAFLD should be maintained.

## 5. Conclusions

In summary, the present study has shown that AO extract may confer pleiotropic effects against a large spectrum of pathological aspects related to NAFLD without side effects, such as weight gain, hepatotoxicity, or hypercholesterolemia, in mice fed with WD. In addition, this study offers insight into the underlying mechanisms of the disease, including the regulation of hepatic adiponectin and FXR activity, by which AO extract ameliorates NAFLD. These beneficial actions and signaling pathways of AO extract appear to support its promising potential as a main or add-on therapeutic regimen to treat NAFLD, as well as lower the risk of comorbidities and exacerbations of NAFLD, although further evidence is needed for the extract’s validation.

## Figures and Tables

**Table 1:** Retention time (Rt), precursor ion, molecule weight, and UV Maxima (λ max) of the identified peaks of the AO extract.

No	Rt (min)	Identification	Molecular Formula	Ionization Mode	Observed Mass (*m/z*)	Mass Difference (mmu)
1	4.7	Tryptophan	C_11_H_12_N_2_O_2_	Positive	159.08491 [M-H_2_O-CO+H]^+^ 188.07128 [M-NH_3_+H]^+^ ^#^ 205.09845 [M+H]^+^	−7.31 0.13 0.74
				Negative	203.07321 [M-H]^−^ 225.05624 [M+Na-2H]^−^	−8.84 −7.75
2	5.4	Adenosine	C_10_H_13_N_5_O_4_	Positive	^#^ 268.10469 [M+H]^+^ 290.08099 [M+Na]^+^ 309.12944 [M+CH_3_CN+H]^+^ 331.11222 [M+CH_3_CN+Na]^+^	0.11 −5.53 −1.69 −0.85
				Negative	266.08804 [M-H]^−^ 380.09201 [M+CF_3_COOH-H]^−^	−0.89 10.22
3	9.5	Unknown	C_26_H_30_O_8_	Positive	471.20419 [M+H]^+^ 493.19201 [M+Na]^+^	2.29 8.18
				Negative	469.17523 [M-H]^−^	−11.02
4	12.8	Unknown	C_30_H_46_O_5_	Positive	^#^ 487.34498 [M+H]^+^ ^#^ 509.32263 [M+Na]^+^ 550.34522 [M+CH_3_CN+Na]^+^	2.64 −1.66 −5.62
				Negative	467.32944 [M-H_2_O-H]^−^ 485.33938 [M-H]^−^ 531.34098 [M+HCOOH-H]^−^	13.30 12.68 8.80
5	14.2	Alisol C 23-acetate	C_32_H_48_O_6_	Positive	^#^ 529.34650 [M+H]^+^ 551.33350 [M+Na]^+^ 592.35418 [M+CH_3_CN+Na]^+^	−6.41 −1.35 −7.23
				Negative	ND	
6	16.8	* Alisol B		Positive	490.38761 [M+NH_4_]^+^ 495.34093 [M+Na]^+^ 536.36193 [M+CH_3_CN+Na]^+^	−2.02 −4.10 −9.65
7	18.2	* Alisol B acetate		Positive	515.36592 [M+H]^+^ 537.34832 [M+Na]^+^ 578.38175 [M+CH_3_CN+Na]^+^	−7.73 −7.27 −0.39

* Compared with reference compounds; ^#^ Observed in orifice 1 voltage of 40 V.

**Table 2:** Histological findings of liver tissues at the end of the study.

Test	Control Group	WD Group	AO (100 mg/kg)	AO (250 mg/kg)
Steatosis (0–3)	0.27 ± 0.19	2.59 ± 0.21 ***	2.47 ± 0.27	1.53 ± 0.23 ^#^
Lobular inflammation (0–3)	0.13 ± 0.08	2.54 ± 0.21 ***	2.33 ± 0.32	1.40 ± 0.36
Ballooning (0–2)	0.40 ± 0.19	1.92 ± 0.08 ***	1.83 ± 0.11	1.61 ± 0.23
NAS (0–8)	0.80 ± 0.43	7.05 ± 0.44 ***	6.64 ± 0.41	4.47 ± 0.37 ^##^
Fibrosis stage (0–4)	0.42 ± 0.21	3.53 ± 0.23 ***	2.56 ± 0.29 ^#^	1.89 ± 0.11 ^###^

All data are expressed as the mean ± S.E.M. and analyzed by a one-way ANOVA. *** *p* < 0.001 vs. control group; ^#^ *p* < 0.05, ^##^ *p* < 0.01, ^###^ *p* < 0.001 vs. WD-induced group.

## Data Availability

Not applicable.

## Supplementary Materials

The following supporting information can be downloaded at: https://www.mdpi.com/article/10.3390/nu14030695/s1, Figure S1: Effects of AO (250 mg/kg) on 24-week duration WD administrated mice.
