# Supplementary material for: Beneficial Activities of Alisma orientale Extract in a Western Diet-Induced Murine Non-Alcoholic Steatohepatitis and Related Fibrosis Model via Regulation of the Hepatic Adiponectin and Farnesoid X Receptor Pathways
Source: Nutrients. 2022 Feb 7;14(3):695. doi: 10.3390/nu14030695 (PMC8839158; doi:10.3390/nu14030695)
Supplement: Supplementary file 1 [file nutrients-14-00695-s001.zip › nutrients-1570806-supplementary.pdf]

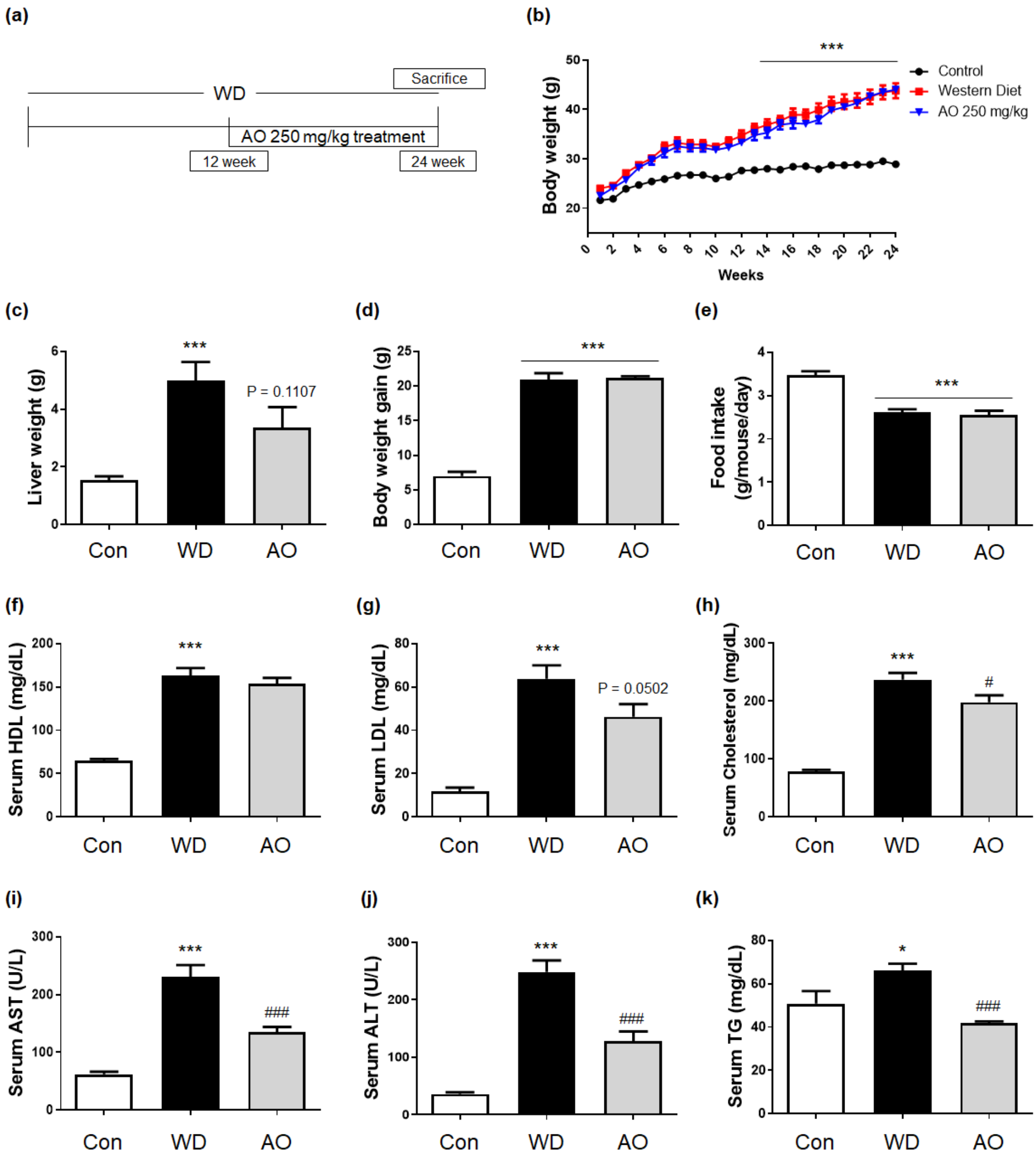

Supplementary Figure S1. Effects of Alisma orientale extract (AO, 250 mg/kg) on 24-week duration western-diet (WD) administrated mice. AO was treated from 12-week to 24-weeks. (a) Scheme of the experiment. (b) The body weight changes in mice following WD-induced. (c) The liver weight and (d) body weight gain were measured. (e) The food intake of mice during the trial was measured. The serum levels of (f) high-density lipoprotein (HDL), (g) low-density lipoprotein (LDL), (h) cholesterol, (i) aspartate transaminase (AST), (j) alanine transaminase (ALT), and (k) triglycerides (TG) were analyzed. * $p < 0.05$, *** $p < 0.001$ vs control group; $^{\#}$ $p < 0.05$, $^{\#\#\#}$ $p < 0.001$ vs WD-treated group ($n = 12$ per group)
